# Assessment of the Psychometric Properties of the Tampa Scale of Kinesiophobia (TSK) Questionnaire in Poland Based on Patients with Type 2 Diabetes Complicated by Stroke

**DOI:** 10.3390/jcm14051751

**Published:** 2025-03-05

**Authors:** Ewelina Bąk, Wojciech Kustrzycki, Robert Skalik, Sylwia Krzemińska

**Affiliations:** 1 Faculty of Health Sciences, University of Bielsko-Biala, 43-300 Bielsko-Biala, Poland; 2 Faculty of Medicine, Wrocław University of Science and Technology, 50-370 Wrocław, Poland; 3 Faculty of Health of Sciences, Higher Medical School in Kłodzko, 57-300 Kłodzko, Poland

**Keywords:** stroke, diabetes, kinesiophobia

## Abstract

**Background/Objectives:** Kinesiophobia, or the fear of movement, is a significant problem in the rehabilitation of patients after a stroke, especially in individuals with diabetes, who have an increased risk of health complications. The aim of the study was to validate the Tampa Scale for Kinesiophobia (TSK) for assessing kinesiophobia in the context of patients with diabetes complicated by stroke to ensure its adequacy and reliability in this specific group of patients. **Methods:** After considering exclusion criteria, 166 patients with type 2 diabetes after ischemic stroke, hospitalized in the neurological rehabilitation ward, were included in the analysis. A survey using the TSK was conducted in the study group. A reliability analysis of the questionnaire was conducted, and then exploratory factor analysis (EFA) and confirmatory factor analysis (CFA) were used to disclose the number of factors that characterize the study group. **Results:** The Cronbach’s alpha value for the entire scale is 0.875. The value for all the questions on the scale was also above 0.86, so they are considered reliable. Removing any question does not increase the value of Cronbach’s alpha or Guttman index. Based on the scree plot, two factors were identified. The first factor includes 12 items and forms a physical factor, while the second factor includes 5 items and forms a psychological factor. The fit of the two-factor model was checked using confirmatory factor analysis. The final two-factor model has an acceptable fit. All the factor loadings are statistically significant. The factor loadings range from 0.262 to 0.729 for the physical factor and from 0.543 to 0.822 for the psychological factor. **Conclusions:** The TSK is a reliable and valid tool for assessing the level of kinesiophobia in a group of patients with type 2 diabetes complicated by stroke. The results of the study using this tool may contribute to the development of more effective therapeutic strategies that take into account the specific physical and psychological needs of this group of patients.

## 1. Introduction

Stroke and diabetes are two conditions that have many common links and contribute to the increasing burden and mortality worldwide. Both the incidence of diabetes and stroke are rising, as they are associated with vascular risk factors such as hypertension and lipid disorders. Diabetes is an established risk factor for stroke and is associated with poorer outcomes after stroke [1]. Abnormal carbohydrate metabolism, which diabetes manifests, occurs in up to two-thirds of people suffering from acute stroke and increases mortality or causes greater disability in patients [1] and may be a contraindication to early stroke therapy [2]. On the other hand, cerebrovascular complications make patients with diabetes 2–6 times more susceptible to stroke, and this risk is exacerbated in younger individuals and patients with hypertension and vascular complications [3,4,5].

The incidence of type 2 diabetes (T2D) is increasing worldwide. This condition depends on various causes: aging of the population, economic development of industrialized countries, and resulting changes in dietary habits and lifestyle. About 537 million adults aged 20 to 79 have diabetes, and it is predicted that this number will rise to 643 million by 2030 and 783 million by 2045 [6]. However, due to the lack of symptoms in the early stages of the disease, T2D is diagnosed when complications such as angina pectoris, myocardial infarction, heart failure, kidney failure, or stroke are already present [6].

The term ‘kinesiophobia’ was first used to describe the fear of movement due to lower back pain [7]. These authors defined this fear as excessive, irrational, and detrimental to health due to reduced activity. Kinesiophobia, or the fear of movement, is a significant problem in the rehabilitation of stroke patients, especially in individuals with diabetes, who have an increased risk of health complications. The TSK tool is commonly used to assess the level of kinesiophobia in patients with various conditions.

The universality of the definition of kinesiophobia has led to its application to many other diseases. The TSK is a tool designed to measure the problem of fear of movement. The significance of the problem of reduced physical activity in the prevention and treatment of many diseases has resulted in numerous adaptations of the diagnostic tool, both in terms of disease and for linguistic and cultural versions [8,9,10,11].

In relation to nervous system diseases, the literature reports the use of the TSK to assess the occurrence of kinesiophobia in multiple sclerosis, Parkinson’s disease, or after a stroke, but to the authors’ knowledge, studies have not been conducted in the specific group of patients with diabetes. The importance of the problem seems significant, if only due to the additional difficulties in the rehabilitation and functioning of patients with diabetes after a stroke due to coexisting neuropathic complications. The tool has not been studied for reliability in this group of patients, which was the inspiration for undertaking this action.

The aim of this study is to validate the TSK tool in the context of patients with diabetes after a stroke to ensure its adequacy and reliability in this specific group. Validating the tool will allow for a better understanding of the level of fear of movement in this population and the development of more effective therapeutic strategies.

## 2. Materials and Methods

### 2.1. Organization of the Study

The study was conducted from January to December 2024. The research included patients of the Neurological Rehabilitation Department of the Railway Hospital in Wilkowice-Bystra in Poland. The study group included 166 patients with type 2 diabetes, after an ischemic stroke, who were admitted for early neurological rehabilitation. Of all 244 patients hospitalized in this period at the Neurological Rehabilitation Department, 179 patients who met the eligibility criteria for the study were qualified (those with type 2 diabetes for at least 9 years and after an ischemic stroke). The patients independently completed the TSK questionnaire, and sociodemographic and clinical data were obtained from medical records.

**Figure 1:**
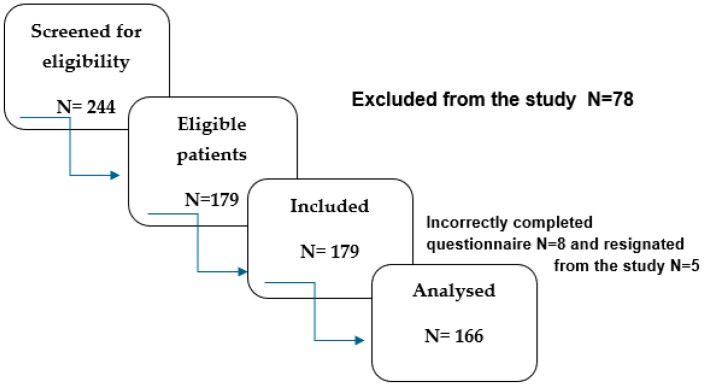
Recruitment process.

The recruitment process is presented in Figure 1.

It is estimated that there are approximately 3.5 million people with diabetes in Poland, which constitutes 9% of the entire population. T2DM (type 2 diabetes mellitus) has been diagnosed in 2 million patients, which constitutes 6% of the entire population. Considering the fact that 6% of the population in Poland has T2DM, it was estimated that the sample should include at least 163 people. Therefore, the sample size used in the study was considered sufficient.

### 2.2. Inclusion and Exclusion Criteria

The following inclusion criteria were applied: age > 18 years, at least 9 years since the diagnosis of type 2 diabetes, no diagnosed cognitive impairments, and patient consent to participate in the study. Exclusion criteria: age < 18 years, less than 9 years since the diagnosis of type 2 diabetes, diagnosed cognitive impairment, and lack of consent to participate in the study.

### 2.3. Tampa Scale for Kinesiophobia

TSK was used in the study. This is a tool consisting of 17 statements (items) and is used with patients to measure fear of movement due to the possibility of experiencing pain. Each item was assigned a 4-point Likert scale (1 = “I do not agree at all”, 2 = “I do not agree in part”, 3 = “I agree partially”, and 4 = “I agree completely”). The minimum score was 17 and the maximum score was 68, with higher scores indicating greater severity of kinesiophobia. The tool has also received numerous adaptations and validations, including Swedish [12], Spanish [13], Portuguese [14], and Japanese [15] versions. The tool was previously translated and linguistically adapted according to international guidelines by authors from Poland [16,17] to conduct research on another group of patients. The instrument was obtained from a publicly available publication.

### 2.4. Statistical Methods

All statistical calculations were performed using the R statistical package version 4.4.2. Variables measured on a quantitative scale were characterized using the mean, standard deviation, and quartiles, while variables measured on a nominal scale were presented using frequencies and percentages. First, the analysis of each item was carried out by calculating the floor effect and the ceiling effect, i.e., the determination of the lowest and highest possible score by the studied group. The internal consistency of a set of items was assessed using Cronbach’s α. Exploratory factor analysis (EFA) and confirmatory factor analysis (CFA) were used in this article to reduce the number of variables to explain a large set of variables and the identification of relationships between the observed variables and latent factors. CFA checked the fit of the model obtained in EFA.

### 2.5. Participants

The study group consisted of a total of 166 people, including 82 women (49.4%) and 84 men (50.6%). Most participants had vocational education n = 67 (40.4%) or secondary education n = 59 (35.5%) and were in relationships n = 115 (69.3%). The majority of the patients were retired n = 101 (60.8%) and lived with their families n = 138 (83.1%) in rural areas n = 88 (53.0%) (Table 1).

All the patients were diagnosed with peripheral neuropathy which was confirmed in the medical records. The average stroke duration (months) was 1.9 ± 0.7. The average value of points on the NIHSS scale was 6 ± 8.4 which indicates moderate severity of stroke symptoms. In the Bartel index, the mean point was 59.5 ± 39.3 (moderate disability), and the mean point on the Rankin Scale was 2.54 ± 1.8 (medium degree of disability). The average duration of diabetes was 11.0 ± 1.5 years, HbA1c level was 10.9 ± 1.4, and BMI was 28.7 ± 4.8. (Table 2).

## 3. Results

### 3.1. Floor and Ceiling Effect

To evaluate the research tool in the study group, the floor and ceiling effects were calculated, taking into account the percentage of respondents who scored below the lower (floor) and upper (ceiling) cut-off points. The cut-off points were set at 5% and 95% of the total TSK score (17–68 points). For the TSK, the lower cut-off was set at 20 points, and the upper cut-off at 65 points. The floor effect is defined if more than 20% of the respondents scored below the lower cut-off, while the ceiling effect is defined if more than 20% of the respondents scored above the upper cut-off. No floor or ceiling effects were observed, as only one person scored below 20 points and one person scored above 65 points. On average, the patients scored 44.6 ± 7.9 points on the TSK (Table 3).

For individual questions, the floor effect was calculated as the percentage of respondents who chose the answer ‘strongly disagree’, while the ceiling effect represents the percentage of respondents who chose the highest score for a given question, i.e., the answer ‘strongly agree’. The floor effect was detected in question 1 (‘I am afraid I might injure myself if I exercise’.) because more than 20% of the people chose the ‘strongly disagree’ option. The ceiling effect was detected in question 12 (‘Although my pain is bothersome, I will feel better if I am physically active’.) because more than 20% of the people chose the ‘strongly agree’ option. Detailed data are presented in Table 4.

### 3.2. Reliability Analysis

The internal consistency of a set of items in the questionnaire was evaluated using Cronbach’s alpha coefficient and Guttman’s index. Values approaching one indicate high reliability of the scale, so we can assume that the responses to individual questions are closely related. The value of Cronbach’s alpha is 0.875. All the questions on the scale were above 0.86, so they are considered reliable (Table 5). Removing any question does not increase the value of Cronbach’s alpha or Guttman’s index. The question most correlated with the scale is “Being cautious, meaning not making any unnecessary movements, is the safest thing I can do to prevent the pain from getting worse”, and the least correlated question is “Although my pain is bothersome, I will feel better if I am physically active”.

Before conducting the exploratory factor analysis (EFA), the suitability of the data was assessed using the Kaiser–Meyer–Olkin (KMO) measure of sampling adequacy and Bartlett’s test of sphericity. The measure of sampling adequacy (MSA) for each individual variable was also calculated. Based on the MSA values, individual variables can be eliminated from the study if the index value is less than 0.5.

### 3.3. Exploratory Factor Analysis

**Figure 2:**
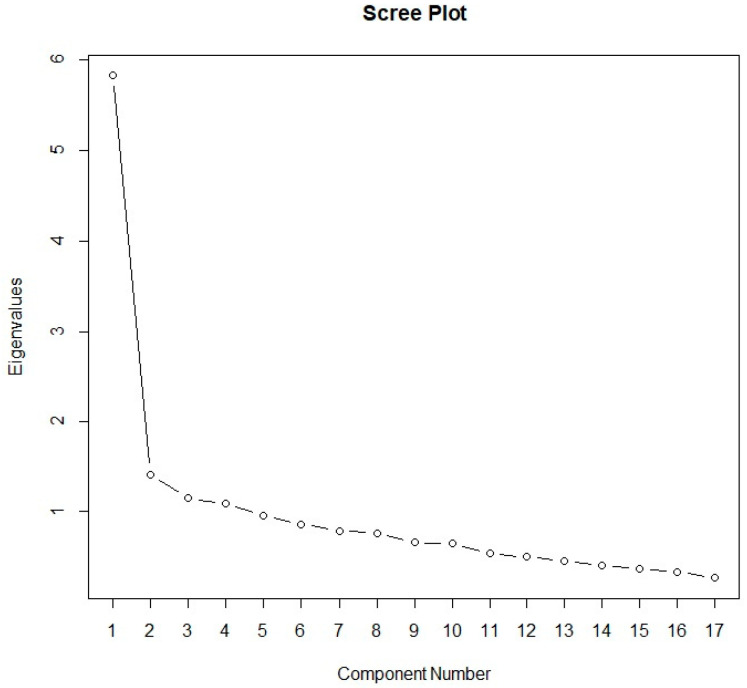
Scree plot for exploratory factor analysis.

Before conducting EFA, it was checked whether all the questions should be included in further analysis. The KMO test result was 0.864, which is quite high, while Bartlett’s test of sphericity showed statistical significance (χ^2^ = 883.71, *p* < 0.0001). The MSA values also indicate that no question can be omitted from further analysis, as the MSA value is greater than 0.5. To determine the number of factors, two of the most popular criteria were used: the eigenvalue criterion and the scree plot criterion. Factor analysis according to the eigenvalue criterion revealed four factors (eigenvalues greater than one). These factors explain a total of 55.7% of the variance of all the variables. The individual variable values were: 1—34.2%, 2—8.3%, 3—6.7%, and 4—6.4%. Based on the scree plot (Figure 2), two factors were identified, and the membership of each factor is specified in Table 6. This table shows the factors with factor loadings greater than 0.3. To better interpret the results, Varimax rotation was performed.

Within the first factor, there are 12 items (T4, T6, T7, T8, T9, T10, T11, T12, T13, T14, T15, and T16), which form the physical factor, while within the second factor, there are 5 items (T1, T2, T3, T5, and T17), which form the psychological factor. The statement most correlated with the psychological factor is ‘If I tried to overcome this fear, the pain would get worse’ (0.827). The statement least correlated with the physical factor is: ‘Although my pain is bothersome, I will feel better if I am physically active’ (0.360) (Table 6).

### 3.4. Confirmatory Factor Analysis

After the EFA, a CFA was estimated. To assess the fit of the CFA, the chi-square test, Root Mean Square Error of Approximation (RMSEA), Comparative Fit Index (CFI), Tucker–Lewis index (TLI), and Standardized Root Mean Squared Residual (SRMR) were used. Given that the questionnaire items are measured on an ordinal scale, the parameters were evaluated using the Diagonally Weighted Least Squares method.

**Figure 3:**
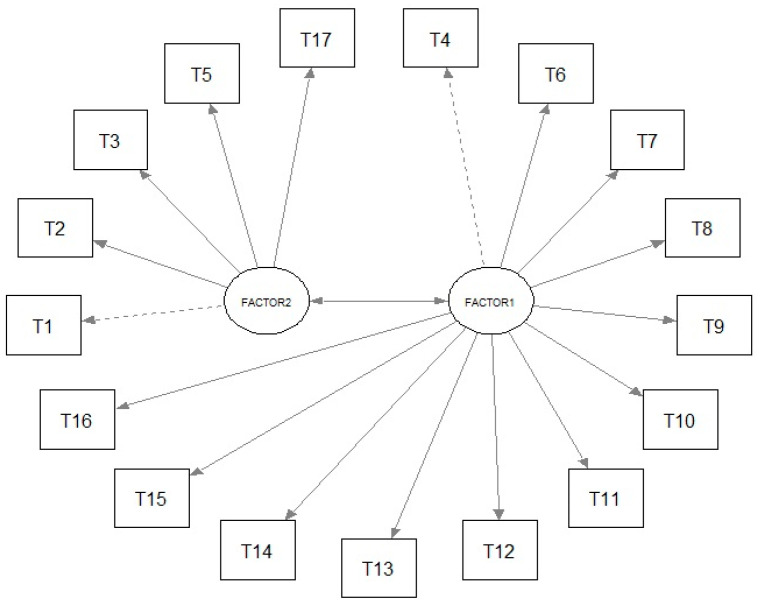
Graphical representation of factor loadings.

The fit of the two-factor model was assessed using CFA. The final two-factor model has an acceptable fit, as indicated by the following indices: χ^2^ (202.218, df = 118, *p* < 0.001), CFI = 0.982, TLI = 0.979, SRMR = 0.073, and RMSEA = 0.066 (90% CI: 0.050–0.081). Figure 3 presents a graphical representation of the model.

The factor loadings of the two-factor model are presented in Table 7 and Figure 3. It should be noted that all the factor loadings are statistically significant. The factor loadings range from 0.262 (T12) to 0.729 (T10) for the physical factor and from 0.543 (T17) to 0.822 (T3) for the psychological factor. The statement with the greatest impact on the psychological factor is “My body tells me that something dangerous is happening to me”, while for the physical factor, it is “Being cautious by not making any unnecessary movements is the safest thing I can do to prevent the pain from getting worse”.

## 4. Discussion

The prevalence of the problem related to the fear of movement and its significance meant that kinesiophobia has been and continues to be studied in an increasingly wide range of disease entities associated with patients experiencing pain and musculoskeletal dysfunction [16,18,19,20,21]. In relation to neurological diseases, studies assessing kinesiophobia have been conducted among patients with Parkinson’s disease [22], multiple sclerosis [23], hemiplegia [24], polish patients with cervical discopathy [25], and elderly Polish patients after stroke [26]. However, there are few studies, this issue is still poorly understood, and according to the authors’ knowledge, no studies on kinesiophobia in patients with T2D complicated by stroke have been conducted so far. This situation motivated the authors to undertake research in this area, and the aim of the study was to adapt and test the psychometric properties of the Polish version of the TSK questionnaire for patients with diabetes complicated by stroke. As is widely known, diabetes as a chronic disease, if not properly treated, leads to serious micro- and macrovascular complications, including stroke, which occurs more frequently in this patient group than in the general population [3,4,5].

As previously mentioned, studies on the psychometric properties of the TSK in the group of patients with T2D complicated by stroke have not been conducted, so the only reference point is the analysis of the tool’s reliability and model divisions. In studies by other authors describing the psychometric properties of the TSK, several different models (one-, two-, three-, and four-factor) were identified depending on the analyzed disease entity [27]. The first reports on the TSK for patients with pain indicated a one-factor structure [28], while subsequent studies analyzed various models adapted to the studied patients [11,12,29]. In the authors’ study, in the group of patients with T2D complicated by stroke, a two-factor structure was identified and confirmed, divided into a physical factor, which included 12 items, and a psychological factor, which included 5 questions with statistically significant factor loadings ranging from 0.262 (T12) to 0.729 (T10) for the physical factor and from 0.543 (T17) to 0.822 (T3) for the psychological factor. The analysis showed that the reliability of both the entire TSK and its individual components was at a good level. The Cronbach’s alpha value for the entire tool is 0.875. The values for all the questions in the scale were also above 0.86, so they are considered reliable. Floor and ceiling effects were found only in individual questions, indicating the construct validity of the test concerning the studied group of patients. In our analysis, higher Cronbach’s alpha values were obtained for the entire tool than in previous studies concerning other patient groups: the Finnish general population (0.72) [11], Japanese patients with back pain (0.85) [15], German patients with back pain (0.82) [30], and Portuguese patients with chronic musculoskeletal pain (0.820) [15]. Importantly, the values from this study in the group of Polish patients with T2D were almost equal to the studies conducted in the group of Polish patients with coronary artery disease (0.878) [16] and Polish patients with pain in cancer (0.84) [17].

The study of kinesiophobia is rarely conducted among Polish patients, and it has not been carried out among patients with T2D so far, which constitutes an innovative contribution to the further development of science. In future studies in this group of patients, attention should be paid to accurately determining the type and severity of coexisting neuropathy, which may affect the occurrence of more severe kinesiophobia, as well as the pain treatment used, which may influence kinesiophobia. The strength of the current study is that there have been no studies in this group of patients so far; however, the authors note some shortcomings that positively indicate new directions for research.

## 5. Conclusions

The Tampa Scale is a reliable and valid tool for assessing the level of kinesiophobia in a group of patients with type 2 diabetes complicated by stroke. The results obtained using this tool can contribute to the development of more effective therapeutic strategies that address the specific needs of this patient group.

### 5.1. Limitations of the Study

The lack of previous studies in this specific patient group makes it difficult to compare results. The study was conducted over one year in a single rehabilitation facility, which may not account for long-term changes in the level of kinesiophobia.

### 5.2. Practical Implications

By assessing the level of kinesiophobia in this group of patients, therapists can tailor rehabilitation programs to the individual needs of patients, taking into account their fear of movement. Regular use of the TSK can help monitor changes in the level of kinesiophobia during therapy. This allows for the evaluation of intervention effectiveness and the introduction of necessary modifications to the treatment plan. Awareness of the level of kinesiophobia can help patients understand their fear and work on overcoming it. This can increase their engagement in the rehabilitation process and improve treatment outcomes. Improvement in quality of life: reducing kinesiophobia can lead to increased physical activity, which in turn can improve the overall quality of life for patients with type 2 diabetes complicated by stroke. Regular physical activity can contribute to better diabetes management and reduce the risk of further complications.

## Figures and Tables

**Table 1:** Sociodemographic data.

Parameter			Total N = 166
Gender	Female		82 (49.4%)
	Male		84 (50.6%)
Age	<60 years		50 (30.1%)
	60–69 years		54 (32.6%)
	70 years and older		62 (37.3%)
Education	Primary and vocational	Primary	17 (10.2%)
		Vocational	67 (40.4%)
	Secondary and higher	Secondary	59 (35.5%)
		Higher	23 (13.9%)
Employment status	Yes	Employed	41 (24.7%)
	No	Unemployed	11 (6.6%)
		Retired	101 (60.8%)
		On disability pension	13 (7.8%)
Marital status	Married		115 (69.3%)
	Other	Single	10 (6.0%)
		Vidived	28 (16.9%)
		Divorced	13 (7.8%)
Place of residence	City		78 (47.0%)
	Village		88 (53.0%)
Living arrangement	Alone		28 (16.9%)
	With family		138 (83.1%)

**Table 2:** Clinical data.

Parameter		Total N = 166
Stroke duration (months)	Mean ± SD	1.9 ± 0.7
	Median	2.0
	Quartiles (25–75%)	1.0–2.0
Rankin Scale	Mean ± SD	2.54 ± 1.8
	Median	3.0
	Quartiles (25–75%)	1.0–4.0
Bartel Index	Mean ± SD	59.5 ± 39.3
	Median	62.5
	Quartiles (25–75%)	20–100
NIHSS score	Mean ± SD	6 ± 8.4
	Median	3.0
	Quartiles (25–75%)	1.0–7.0
Duration of diabetes (years)	Mean ± SD	11.0 ± 1.5
	Median	11.0
	Quartiles (25–75%)	10.0–12.0
Fasting glucose	Mean ± SD	138.7 ± 7.8
	Median	141.5
	Quartiles (25–75%)	135.0–143.0
HbA1C	Mean ± SD	10.9 ± 1.4
	Median	11.2
	Quartiles (25–75%)	9.8–12.2
BMI	Mean ± SD	28.7 ± 4.8
	Median	28.0
	Quartiles (25–75%)	25.3–31.2

**Table 3:** Average for TSK.

	N	M	SD	Min	Max	Q1	Me	Q3
TSK	166	44.6	7.9	17.0	67.0	41.0	45.0	49.0

Note: M—mean; SD—standard deviation; Q1—25% quartile; Me—median; Q3—75% quartile; Min—minimum value; Max—maximum value.

**Table 4:** Floor and ceiling effect.

	TSK	
Question	Floor Effect	Ceiling Effect
1	41 (24.7%)	14 (8.4%)
2	30 (18.1%)	8 (4.8%)
3	11 (6.6%)	33 (19.9%)
4	11 (6.6%)	24 (14.4%)
5	18 (10.8%)	17 (10.2%)
6	16 (9.6%)	29 (17.5%)
7	8 (4.8%)	25 (15.1%)
8	10 (6.0%)	14 (8.4%)
9	8 (4.8%)	18 (10.8%)
10	14 (8.4%)	24 (14.4%)
11	11 (6.6%)	18 (10.8%)
12	5 (3.0%)	53 (31.9%)
13	8 (4.8%)	33 (19.9%)
14	18 (10.8%)	16 (9.6%)
15	9 (5.4%)	26 (15.7%)
16	12 (7.2%)	14 (8.4%)
17	17 (10.2%)	16 (9.6%)

**Table 5:** Reliability analysis for the questionnaire measuring the level of kinesiophobia.

**Reliability Statistics**			**Cronbach’s Alpha**	0.875	**Guttman Index**	**0.893**
	**95% Confidence Interval**		**Scale Statistics:**	Mean ± SD	N of Item	
	lower	upper		2.62 ± 0.46	17	
	0.847	0.902				
Items	Statements			Mean ± SD	Corrected Item-Total Correlation	Cronbach’s Alpha if Item Deleted
T1	I am afraid I might injure myself if I exercise.			2.27 ± 0.93	0.529	0.869
T2	If I tried to overcome this fear, the pain would get worse.			2.24 ± 0.80	0.521	0.869
T3	My body tells me that something dangerous is happening.			2.81 ± 0.83	0.653	0.864
T4	My pain would probably decrease if I exercised.			2.72 ± 0.78	0.563	0.867
T5	People do not take my health condition seriously enough.			2.43 ± 0.82	0.496	0.870
T6	My injury has caused my body to be at risk for the rest of my life.			2.66 ± 0.88	0.615	0.865
T7	Pain always means that I have injured my body.			2.72 ± 0.78	0.584	0.867
T8	Just because something increases pain does not mean it is dangerous.			2.64 ± 0.72	0.387	0.874
T9	I am afraid I might accidentally injure myself.			2.67 ± 0.73	0.574	0.867
T10	Being cautious, meaning not making any unnecessary movements, is the safest thing I can do to prevent the pain from getting worse.			2.59 ± 0.84	0.661	0.863
T11	I would not feel this kind of pain if there was nothing potentially dangerous happening in my body.			2.67 ± 0.76	0.644	0.864
T12	Although my pain is bothersome, I will feel better if I am physically active.			3.06 ± 0.80	0.248	0.879
T13	Pain tells me when to stop exercising to avoid injury.			2.83 ± 0.80	0.550	0.868
T14	It is really not safe for someone in my condition to be physically active.			2.39 ± 0.81	0.619	0.868
T15	I cannot do all the things that normal people do because I can easily get injured.			2.78 ± 0.77	0.604	0.866
T16	Even if something causes me a lot of pain, I do not think it is really dangerous.			2.54 ± 0.75	0.575	0.867
T17	No one should exercise if they feel pain.			2.60 ± 0.80	0.448	0.872

**Table 6:** Factor loadings for the two-factor model (exploratory factor analysis).

		Factor 1	Factor 2	Measures of Sampling Adequacy (MSA)
T1	I am afraid I might injure myself if I exercise.	0.106	0.751	0.843
T2	If I tried to overcome this fear, the pain would get worse.	0.039	0.827	0.811
T3	My body tells me that something dangerous is happening.	0.473	0.500	0.868
T4	My pain would probably decrease if I exercised.	0.600	0.171	0.852
T5	People do not take my health condition seriously enough.	0.267	0.512	0.897
T6	My injury has caused my body to be at risk for the rest of my life.	0.537	0.380	0.894
T7	Pain always means that I have injured my body.	0.659	0.125	0.863
T8	Just because something increases pain does not mean it is dangerous.	0.387	0.221	0.825
T9	I am afraid I might accidentally injure myself.	0.611	0.244	0.897
T10	Being cautious, meaning not making any unnecessary movements, is the safest thing I can do to prevent the pain from getting worse.	0.563	0.407	0.867
T11	I would not feel this kind of pain if there was nothing potentially dangerous happening in my body.	0.569	0.344	0.905
T12	Although my pain is bothersome, I will feel better if I am physically active.	0.360	−0.122	0.768
T13	Pain tells me when to stop exercising to avoid injury.	0.672	0.094	0.886
T14	It is really not safe for someone in my condition to be physically active.	0.538	0.310	0.842
T15	I cannot do all the things that normal people do because I can easily get injured.	0.548	0.312	0.820
T16	Even if something causes me a lot of pain, I do not think it is really dangerous.	0.700	0.065	0.900
T17	No one should exercise if they feel pain.	0.114	0.663	0.860

**Table 7:** Standardized factor loadings (FLs) for the two-factor model.

		FL	SE	FL/SE	p
Factor 1 (Physical Factor)					
T4	My pain would probably decrease if I exercised.	0.622	0.027	23,217	<0.001
T6	My injury has caused my body to be at risk for the rest of my life.	0.678	0.026	25,776	<0.001
T7	Pain always means that I have sustained a bodily injury.	0.640	0.026	24,246	<0.001
T8	Just because something increases pain doesn’t mean it is dangerous.	0.414	0.027	15,078	<0.001
T9	I am afraid that I might accidentally injure myself.	0.638	0.026	24,501	<0.001
T10	Being cautious by not making any unnecessary movements is the safest thing I can do to prevent the pain from getting worse.	0.729	0.027	27,318	<0.001
T11	I wouldn’t feel such pain if there wasn’t something potentially dangerous happening in my body.	0.717	0.026	27,400	<0.001
T12	Although my pain is bothersome, I will feel better if I stay physically active.	0.262	0.029	8,998	<0.001
T13	Pain tells me when to stop exercising to avoid injury.	0.611	0.026	23,372	<0.001
T14	It really isn’t safe for someone in my health condition to be physically active.	0.685	0.028	24,520	<0.001
T15	I can’t do all the activities that normal people do because I can easily get injured.	0.674	0.027	24,552	<0.001
T16	Even if something causes me a lot of pain, I don’t think it is really dangerous.	0.631	0.028	22,648	<0.001
Factor 2 (Psychological Factor)					
T1	I am afraid that I might get injured if I exercise.	0.673	0.032	20,955	<0.001
T2	If I tried to overcome this fear, the pain would get worse.	0.658	0.032	20,893	<0.001
T3	My body tells me that something dangerous is happening to me.	0.822	0.034	23,960	<0.001
T5	People do not take my health condition seriously enough.	0.588	0.031	19,090	<0.001
T17	No one should exercise if they feel pain.	0.543	0.033	16,610	<0.001

FL—factor loadings; SE—standard error.

## Data Availability

The data can be provided upon reasonable request to the corresponding author.
